# ﻿*Erythroxylumaustroguangdongense* (Erythroxylaceae), a new species from Guangdong, China

**DOI:** 10.3897/phytokeys.202.84688

**Published:** 2022-07-29

**Authors:** Chun-Mei He, Xin-Xin Zhou, Xue-He Ye, Weijun Chen, Yi-Hua Tong

**Affiliations:** 1 Guangdong Provincial Key Laboratory of Silviculture, Protection and Utilization, Guangdong Academy of Forestry, Guangzhou, 510520, China Guangdong Academy of Forestry Guangzhou China; 2 Key Laboratory of Plant Resources Conservation and Sustainable Utilization & Key Laboratory of Digital Botanical Garden of Guangdong Province, South China Botanical Garden, Chinese Academy of Sciences, Guangzhou, 510650, China South China Botanical Garden, Chinese Academy of Sciences Guangzhou China; 3 Zhongkai University of Agriculture and Engineering, Guangzhou, 510225, China Zhongkai University of Agriculture and Engineering Guangzhou China; 4 Zhuhai Charmview International Travel Co. LTD, Zhuhai, 519000, China Zhuhai Charmview International Travel Co. LTD Zhuhai China; 5 Center of Conservation Biology, Core Botanical Gardens, Chinese Academy of Sciences, Guangzhou, 510650, China Center of Conservation Biology, Core Botanical Gardens, Chinese Academy of Sciences Guangzhou China

**Keywords:** Coca family, morphology, taxonomy

## Abstract

*Erythroxylumaustroguangdongense* (Erythroxylaceae), a new species from Guangdong Province, China, is described and illustrated. This new species is morphologically most similar to *E.calyptratum*, but is distinguished by the leathery leaf blade with fewer pairs of secondary veins and flowers borne on leafless nodes of the basal part of the current branch with much longer pedicels and sub-rectangular petal appendages. This is the second native species of *Erythroxylum* recorded from China.

## ﻿Introduction

The genus *Erythroxylum* P. Browne (Erythroxylaceae), with about 264 species, is widely distributed in tropics and subtropics with the center of its diversity in Neotropics (POWO 2022). Many species of this genus contain the substance cocaine, and can be used medicinally as a narcotic. In China, only two species of *Erythroxylum* are recorded, i.e., the introduced *E.novogranatense* (D. Morris) Hieron. and the widely distributed *E.sinense* Y. C. Wu (Liu and Bartholomew 2008).

During field surveys of medicinal plant resources in Guangdong Province, we encountered an unknown *Erythroxylum* species with white flowers, while the species previously recorded from China either has whitish-yellow (*E.novogranatense*) or pinkish (*E.sinense*) flowers. After a morphological comparison with specimens in the herbarium (IBSC) and consulting the relevant literature (e.g. Guillaumin 1907; Chung 1996; Hô 2003; Liu and Bartholomew 2008; Harwood and Chayamarit 2011; Komada et al. 2018), we concluded that this unknown species is morphologically distinct from all the other species previously known in China and its surrounding countries. Thus, we describe and illustrate it as a new species.

## ﻿Materials and methods

Flowering and fruiting material was collected from Zhuhai and Taishan, Guangdong Province, China during several field trips from 2019 to 2022. Descriptions were based on both living and dried collections, which were deposited at the herbarium of South China Botanical Garden, Chinese Academy of Sciences (**IBSC**). Measurements were performed with a ruler, and small plant parts were observed and measured under a stereo microscope (Mshot-MZ101).

## ﻿Taxonomic treatment

### ﻿ Erythroxylum austroguangdongense

Taxon classificationPlantaeMalpighialesErythroxylaceae

C. M. He, X. X. Zhou & Y. H. Tong sp. nov.

B08D3E51-1F9A-500C-A688-A886BB63FE73

urn:lsid:ipni.org:names:77302515-1

Fig. 1

#### Type.

China. Guangdong Province: Taishan, Chaliao Ao, 22°12'54.87"N, 112°57'34.84"E, 563 m a.s.l., 3 April 2021 (fl.), *Xin-Xin Zhou & Yue-Yao Liu LSX303* (holotype: IBSC, isotypes: IBSC).

#### Diagnosis.

Most similar to *E.calyptratum* Komada & Tagane in having reddish brown to grayish brown branches with dense lenticels, and white petals with appendages, but distinguished by the leathery (vs. thinly chartaceous) leaf blade with fewer pairs of secondary veins (6–8 pairs vs. 11–15 pairs), and flowers borne on leafless nodes of the basal part of current branch (vs. in leaf axils) with longer pedicels (1–1.5 cm vs. 5.2–7 mm) and sub-rectangular petal appendage (vs. bilobed appendage with each lobe consisting of a short anterior auricle and a large posterior auricle). A more detailed comparison of the two species is shown in Table 1.

**Table 1.** Morphological comparison of *Erythroxylumaustroguangdongense* and *E.calyptratum*.

Characters	*E.austroguangdongense*	*E.calyptratum*
Leaf blade texture	Leathery	Thinly chartaceous
Leaf blade shape	Elliptic or lanceolate	Elliptic, oblong, oblong-lanceolate or ovate
Leaf blade color	Dark green and shining adaxially	Pale green adaxially
Secondary veins	6–8 pairs	11–15 pairs
Flower	Solitary on leafless nodes of the basal part of current branch	Solitary to 3-fascicled in leaf axils
Pedicel length	1–1.5 cm	5.2–7 mm
Petal appendage	Sub-rectangular	Bilobed and each lobe consisting of a short anterior auricle and a large posterior auricle

**Figure 1.**
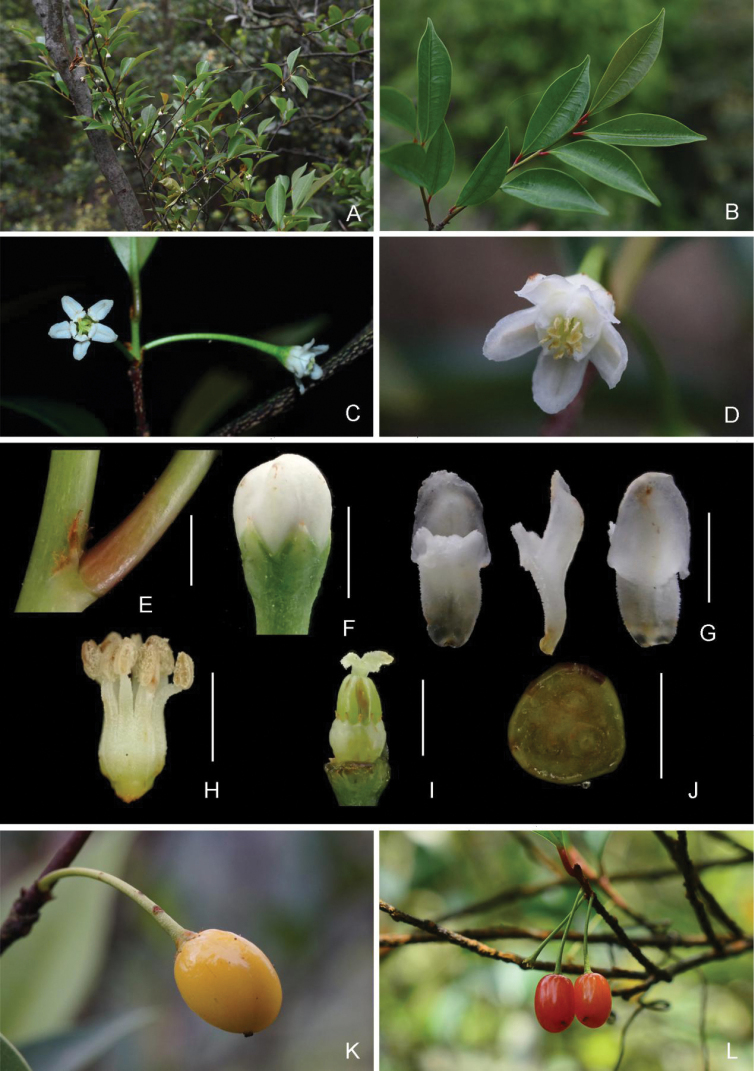
*Erythroxylumaustroguangdongense***A** flowering branches **B** leafy branches **C** female flowers **D** male flower **E** stipule, showing fimbriate margin **F** flower bud, showing calyx **G** petals, adaxial (left), lateral (middle) and abaxial (right) view **H** androecium **I** staminodes and pistil **J** cross section of ovary, showing one fertile locule (low right) **K–L** fruits. Scale bars: 3 mm (**H**); 2 mm (**E–G, I**); 1 mm (**J**). Photographs **A–B** by Xin-Xin Zhou **D, K** by Wei-Jun Chen **H** by Xue-He Ye and others by Chun-Mei He.

#### Description.

Shrubs deciduous, 1.5–2 m tall, dioecious. Young branches greenish, old branches reddish-brown to grayish-brown, lenticellate. Stipule triangular to narrowly triangular, 1.8–3 × 0.7–0.8 mm, margin entire when young, gradually fimbriate, or dissected, caducous when old. Leaves alternate, simple; petiole 3.5–6 mm long; blade elliptic or lanceolate, 4–7.7 × 1.6–2.5 cm, leathery, apex acuminate, acumen to 5 mm long, base cuneate or attenuate, margin entire, dark green and shining adaxially, pale green abaxially, midrib sunken adaxially, prominent and pale yellow to brownish-yellow abaxially, secondary veins 6–8 pairs, almost flat, faintly visible on both surfaces, tertiary veins reticulate, obscure adaxially, slightly visible abaxially. Flowers solitary on leafless nodes of the basal part of current branch, pedicel 1–1.5 cm long; bracteole triangular, ca. 0.8 mm long, margin fimbriate or serrately dissected; calyx 1.6–1.8 mm long, lobes 5, ovate-triangular, ca. 1 mm long, apex brownish, acute; petals 5, white, oblong, 3.7–4 mm long, apex obtuse or rounded, adaxially with one appendage attached ca. 1.5 mm above from base, appendage sub-rectangular, ca. 1.6 × 1 mm, papillate throughout, slightly concave on both upper and lower margin. Stamens or staminodes 10. Male flowers: stamens with different length of filaments arranged alternately, short filaments ca. 3.7 mm long, long ones ca. 4 mm long, all basally connate into a tube, tube ca. 2 mm long, densely covered with papillary trichomes; anthers ca. 1.1 mm long; sterile pistil ca. 4 mm long. Female flowers: staminodes ca. 1.2 mm long, staminodal tube ca. 1 mm long, anther absent; ovary ellipsoidal, 2–2.5 mm long, 1–1.3 mm in diam., 3-locular, with 1 fertile locule, styles 3, entirely free at base, ca. 1 mm long including stigma, stigma clavate, ca. 0.5 mm long, reflexed, with papillae. Young fruits green, turning to yellowish, ripening red, ovoid to reniform, apex oblique, 7–9 × 2.5–3 mm.

#### Etymology.

The species epithet is named after the distribution area of this new species, South Guangdong.

#### Vernacular name.

南粤古柯 (Chinese pinyin: nán yuè gŭ kē).

#### Distribution and habitat.

This species is currently known only from Guangdong Province, China. Three populations are found in Yangchun (E’huangzhang), Zhuhai (Fenghuang Mountain) and Taishan (Gudou Mountain) respectively. It grows in evergreen broadleaved forests at elevations of 170–800 m.

#### Conservation Status.

According to the field observation, the number of mature individuals of *Erythroxylumaustroguangdongense* is less than 100. Thus, it is assigned a status of ‘Endangered’ (EN) following the IUCN Red List categories and criteria (IUCN Standards and Petitions Committee 2022). Since most of its distribution area is under the protection of E’huangzhang Natural Reserve and Gudou Mountain Natural Reserve, and it is not economically valuable, the threat risk seems to be low.

#### Phenology.

Flowering in March-April and fruiting in April-August.

#### Additional specimens examined (paratypes).

China. Guangdong, Taishan: Banyuzui, 448 m a.s.l., 28 March 2019 (fl.), *Lei Jiang*, *Jin-Fan Lin*, *Jin-Ye Feng*, *Fu-Jun Chen & Jie-Lin Chen GDS-00291* (IBSC); Chaliao Ao, 205 m a.s.l., 25 April 2019 (fr.), *Teng-Hui Guo*, *Jin-Fan Lin & Jia-Ming Chen GDS-00484* (IBSC); ibid., 19 May 2019 (fr.), *Taishan Expedition 440781190519024LY* (IBSC). Yangchun: Bajia, Xianjiadong Reservoir, 800 m a.s.l., 1 August 2001 (fr.), *Hua-Gu Ye 6094* (IBSC); Bajia Reservoir, 750 m a.s.l., 5 August 2001 (fr.), *Hua-Gu Ye 6199* (IBSC). Zhuhai: Fenghuang Mountain, 177 m a.s.l., 2 April 2021 (young fr.), *Yi-Hua Tong*, *Wei-Jun Chen & Feng Ling TYH-2526* (IBSC); ibid. 20 March 2022 (fl.), *Wei-Jun Chen TYH-2561* (IBSC).

## ﻿Discussion

This new species is the second native species reported from China, and it differs from previously recorded species, i.e., *Erythroxylumsinense*, in having leathery (vs. chartaceous) leaf blades with faintly visible (vs. prominent) veins and white (vs. pinkish) petals with sub-rectangular (vs. ligule-like) appendages.

*Erythroxylum* species, especially *E.coca* Lam. and *E.novogranatense*, are well known for their tropane alkaloids, such as cocaine (Aynilian et al. 1974). According to Lv et al. (2022), 383 compounds, including diterpenes, triterpenes, flavonoids, alkaloids, and other derivates, have been found in 67 *Erythroxylum* species. Investigating this new taxon for its phytochemical constituents could lead to the discovery of novel sources of these compounds, as well as possibly new compounds unknown to science.

## Supplementary Material

XML Treatment for Erythroxylum austroguangdongense
